# Glycemic Dynamics and Management Strategies in Individuals With Type 1 Diabetes During Ramadan: A Continuous Glucose Monitoring-Based Narrative Review

**DOI:** 10.7759/cureus.116933

**Published:** 2026-09-25

**Authors:** Abubakr Sowilem, Abdelrhman K Mohamed, Emad Wissa, Raof Rashed, Mahmoud M Magdi

**Affiliations:** 1 Endocrinology and Diabetes, Al-Sabah Hospital, Kuwait City, KWT; 2 Internal Medicine, Assiut University Hospitals, Assiut, EGY; 3 Internal Medicine, Al-Sabah Hospital, Kuwait City, KWT; 4 Internal Medicine, Benha University, Benha, EGY; 5 Internal Medicine, Faculty of Medicine, Zagazig University, Zagazig, EGY

**Keywords:** continuous glucose monitoring, glycemic control, pre-ramadan education, ramadan fasting, type 1 diabetes mellitus (t1d)

## Abstract

Ramadan is the ninth month of the Islamic lunar calendar, during which healthy adult Muslims fast daily from sunrise to sunset. Fasting during Ramadan presents unique physiological and behavioral challenges for individuals with type 1 diabetes mellitus (T1DM), who are generally classified as high- to very-high-risk for fasting. Because traditional markers such as hemoglobin A1C (HbA1c) and self-monitored blood glucose fail to capture short-term glycemic excursions, continuous glucose monitoring has become essential for evaluating real-world glycemic dynamics during this period. This review examines recent continuous glucose monitoring-based studies to characterize the direction, magnitude, and determinants of glycemic changes during Ramadan. Across the literature, findings demonstrate considerable heterogeneity, ranging from glycemic deterioration to temporary improvement or stability, influenced heavily by baseline glycemic control, age, insulin delivery modalities, and whether participants received structured pre-Ramadan education. Notably, exposure to hypoglycemia does not appear to increase during Ramadan fasting; instead, postprandial hyperglycemia clustered around the evening and pre-dawn meals emerges as the predominant and most variable challenge. Structured pre-Ramadan education, individualized insulin adjustments, and continuous glucose monitoring-guided management are critical levers for supporting safer fasting in this population.

## Introduction and background

Depending on the location and time of year, people observe Ramadan, the ninth month of the Islamic lunar calendar, by fasting from sunrise to sunset for roughly 11 to 20 hours. A significant percentage of Muslims with type 1 diabetes mellitus (T1DM) opt to fast for religious and cultural reasons, despite the fact that Islamic law exempts people with chronic illnesses, including diabetes, from the requirement to fast. The Epidemiology of Diabetes and Ramadan (EPIDIAR) study estimated that 42.8% of people with T1DM fasted for at least 15 days during Ramadan, with the highest prevalence reported in Saudi Arabia (71.6%). The same study reported a 4.7-fold increase in severe hypoglycemia among people with T1DM during Ramadan compared with non-fasting periods [1]. The International guidelines from the International Diabetes Federation and the Diabetes and Ramadan (DAR) International Alliance continue to classify most people with T1DM as high- to very-high-risk fasters [2].

In the past, HbA1c and self-monitored blood glucose (SMBG) were used to assess glycemic control during Ramadan. Both methods have well-known drawbacks: SMBG provides only isolated point-in-time readings, while HbA1c reflects average glycaemia over approximately three months and cannot capture short-term excursions [3]. By producing time-in-range (TIR), time-above-range (TAR), time below range (TBR), and glycemic variability (GV) metrics over predetermined periods, continuous glucose monitoring (CGM), whether real-time (rt-CGM) or intermittently scanned/flash (isCGM/FGM), fills in these gaps and allows clinicians to describe not only whether glycemic control changes during fasting but also when and why [4].

Four recent, real-world CGM investigations in individuals with T1DM who are fasting throughout Ramadan are summarized in this narrative review: two adult cohorts, one primarily adolescent cohort from Saudi Arabia, and one pediatric/adolescent cohort from Malaysia. The objectives are to describe the direction and causes of CGM-derived glycemic shift during Ramadan, reconcile findings that appear to be inconsistent between research, and identify implications for CGM-supported therapeutic treatment of fasting in type 1 diabetes.

## Review

Methods

This review is narrative rather than systematic. Four studies - Surrati et al. (2026, Scientific Reports) [5], Alguwaihes et al. (2025, Frontiers in Endocrinology) [6], Al Hayek and Al Dawish (2026, Journal of Diabetes Science and Technology) [7], and Teoh et al. (2022, Journal of the ASEAN Federation of Endocrine Societies) [8] were purposefully chosen because they all used objective CGM-derived metrics (rather than SMBG or HbA1c alone) to compare glycemic control in individuals with T1DM. Because the demographics, CGM devices, glycemic thresholds, and statistical methods of the four studies varied greatly, the results are combined thematically rather than via a formal meta-analysis.

Summary of the included studies

Table 1 summarizes the design, population, and CGM methodology of each study. The four studies use FreeStyle Libre-family sensors and cover a wide age range (children as young as eight years old through adults) and two geographic/cultural contexts (Saudi Arabia and Malaysia). However, the scanning modalities (retrospective/professional vs. patient-facing intermittently scanned) and predefined assessment windows vary.

**Table 1:** Summary of the design, population, and continuous glucose monitoring methodology of each study. T1DM: type 1 diabetes mellitus; CGM: continuous glucose monitoring

Study	Country/setting	Population	N	CGM device	Design	Assessment windows
Surrati et al., 2026 [5]	Saudi Arabia (Madinah)	Adults with T1DM (median age: 30 years)	81	FreeStyle Libre 2 (isCGM)	Prospective observational	Pre-Ramadan (14 days); first two weeks and last two weeks of Ramadan 2025
Alguwaihes et al., 2025 [6]	Saudi Arabia (Riyadh)	Adults with T1DM; non-DM age-matched comparators	39 completers (of 76 enrolled); 49 controls	FreeStyle Libre 14-day (FGM)	Three-year prospective observational (2020-2022)	Four weeks before, during, and after Ramadan, each of three years
Al Hayek and Al Dawish, 2026 [7]	Saudi Arabia (Riyadh)	T1DM, mostly adolescents (median age 17 years)	186	FreeStyle Libre 2 (isCGM)	Ambispective (retro + prospective)	One month before, during, and one month after Ramadan 2024
Teoh et al., 2022 [8]	Malaysia (Putrajaya and Kelantan)	Children/adolescents with T1DM (8-18 years)	24	iPro2 retrospective professional CGM	Prospective observational	Before and during Ramadan 2020

Direction of glycemic change during Ramadan: a heterogeneous picture

The question of whether Ramadan fasting worsens, improves, or leaves glycemic control in T1DM constant is not answered by any of the four investigations. Table 2 summarizes the principal CGM findings.

**Table 2:** Summary of the principal continuous glucose monitoring findings, including time in range, time below range, and key modifiers of glycemic outcomes. *The notation p=0.004/0.002 corresponds to the statistical significance for changes in time in range compared to baseline, where p=0.004 represents weeks 1-2, and p=0.002 represents weeks 3-4 of Ramadan. Iftar refers to the evening meal consumed at sunset to break the daily fast. Suhoor refers to the pre-dawn meal consumed before the resumption of the daily fast at sunrise. HbA1c: hemoglobin A1c; SMBG: self-monitored blood glucose

Study	Direction of change during Ramadan	Time in range (TIR)	Time below range (TBR)/hypoglycemia	Key modifiers of outcome
Surrati et al., 2026 [5]	Deterioration	51.5% → 45.0% (weeks 1-2) → 47.0% (weeks 3-4); p=0.004/0.002*	Stable (~3%, no significant change)	Baseline HbA1c >7% showed larger, sustained declines in TIR; HbA1c ≤7% showed small, transient change
Alguwaihes et al., 2025 [6]	Deterioration (progressive across study years)	Not pooled numerically; TIR significantly lower during Ramadan only in 2022 cohort	Lower only in 2022; stable in 2020-2021	Post-COVID lifestyle liberalization (2022); underestimated insulin dose/timing adjustment for Iftar/Suhoor
Al Hayek and Al Dawish, 2026 [7]	Improvement (transient)	59% → 76% during Ramadan (p<0.001); reverted to 56% one month after	Decreased during Ramadan (TBR<54: 2%→0%; TBR54-69: 5%→3%); rose again after	Structured pre-Ramadan education; younger age/adolescents and insulin-pump users had better outcomes
Teoh et al., 2022 [8]	Essentially neutral	51.1% → 42.4% (level 1), p=0.05 (borderline, not significant at strict threshold)	No significant change (level 1 and 2 hypoglycemia stable)	Structured Ramadan-focused education and mandated frequent SMBG; no benefit seen from optimal baseline HbA1c

Evidence of Deterioration

Surrati et al. observed a statistically significant decrease in TIR from 51.5% pre-Ramadan to 45.0% in the first two weeks and 47.0% in the last two weeks, along with rising average glucose concentrations and a slight but significant increase in TAR by the later Ramadan period, in 81 adults with T1DM (median HbA1c 8.0%). Participants with baseline HbA1c >7% (TIR dropping from 46% to 35%) showed significantly higher degradation than those with HbA1c ≤7% (TIR largely preserved, 57% to 54% then back to 57%) [5]. Similar reproducible postprandial hyperglycemic spikes were reported by Alguwaihes et al. in a three-year prospective study of adults in Riyadh following both Iftar (mean difference 15.5 mg/dL) and Suhoor (mean difference 18.8 mg/dL). These abnormal glycemic excursions persisted for at least a month after Ramadan ended. Formal TIR/TAR/TBR differences became statistically significant primarily in the last year of the study (2022), when COVID-19 restrictions were lifted, and more liberal social and dietary patterns were resumed. Additionally, there was a documented increase in insulin dosing, which the authors interpret as proof that insulin adjustments in routine practice had previously been underestimated [6].

Evidence of Transient Improvement

The composite Glycemia Risk Index (GRI) [9] improved significantly during Ramadan, falling from 56.4 pre-Ramadan to 25.6 during fasting - a 54.6% relative reduction - according to the ambispective study of 186 mostly teenage participants (median age = 17 years) by Al Hayek and Al Dawish. Concurrently, the hypoglycemia component (CHypo, 6.0-2.4) and the hyperglycemia component (CHyper, 21.0-12.5) improved, while TIR increased from 59% to 76%. Notably, these improvements were short-lived: a month after Ramadan's conclusion, GRI increased to 53.0, and TIR decreased to 56%, suggesting that the improvement was more directly related to the regimented, streamlined practices of the fasting month than it was to a long-term shift in self-management [7].

Evidence of Glycemic Neutrality

While mean sensor glucose and estimated A1c increased slightly and statistically significantly during Ramadan, Teoh et al.'s smaller pediatric/adolescent cohort (n=24, mean HbA1c 9.6%) in Malaysia found no significant differences in TIR, TAR, TBR, or GV metrics between the pre-Ramadan and Ramadan periods (TIR level 1, 51.1% vs. 42.4%, p=0.05 - borderline but not conventionally significant). Although the baseline HbA1c in this group (9.6%) was greater than in any other examined study, the authors found that fasting itself was not linked to short-term glycemic worsening when supplemented by structured Ramadan-focused instruction and required regular SMBG [8].

Discussion

Rather than just being a limitation of the evidence base, the apparent conflict between these four studies is instructive. Some cohorts declined, one improved, and one stayed steady, which seems to be explained by a number of recurrent causes.

Major Modifier is Structured Education

Teoh et al. [8] and Al Hayek and Al Dawish [7] describe required, structured pre-Ramadan education programs administered by multidisciplinary teams (endocrinologists, diabetes educators, and dietitians), including customized insulin dosage and timing adjustment before the start of the fasting month. Both studies report neutral or improved outcomes. In contrast, Alguwaihes et al. report that only 41-67% of participants over the course of the three study years had documented Ramadan-focused education, and Surrati et al. [5] specifically state that "no structured Ramadan education program was implemented," with care instead provided as individualized counseling during routine clinic visits. This pattern is in line with a large body of previous research (mentioned in the reviewed publications) demonstrating that extensive glucose monitoring, proactive insulin reiteration, and structured instruction can stop or reverse the glycemic decline that would otherwise be linked to fasting.

Age and Pubertal Status

The two cohorts with the best results were also the youngest: Teoh et al.'s [8] participants ranged in age from 8 to 18 years, while Al Hayek and Al Dawish's [7] cohort had a median age of 17 years. Adolescents performed better than adults in the same study (TIR increased from 63% to 76% in adolescents vs. 45% to 70% in adults, with adults exhibiting higher GRI and CHypo throughout). The two adult-only cohorts (Surrati et al. [5], mean age of 30 years; Alguwaihes et al. [6], mean age of 28 years) either demonstrated a net decline or, at worst, a patchy and uneven improvement. This probably reflects a combination of younger patients' higher insulin sensitivity, closer parental/caregiver supervision, and more consistent meal timing. However, Teoh et al.'s own sub-analysis, which showed worse TIR in children under 10 years compared to those over 10 years, also raised the possibility of a more conservative (lower) insulin-dosing approach by clinicians and caregivers of younger children, motivated by hypoglycemia avoidance [8].

Insulin Delivery Modality

When compared to multiple daily injections (MDI), the use of an insulin pump (continuous subcutaneous insulin infusion, or CSII) was consistently linked to superior glycemic results. Insulin pump therapy (IPT) users exhibited higher reductions in GV and lower GRI before (43.0 vs. 62.0) and during Ramadan (23.6 vs. 27.2) compared to MDI users in Al Hayek and Al Dawish's sample. Teoh et al.'s pediatric cohort was nearly entirely MDI-treated (91.7%) and Alguwaihes et al.'s cohort was primarily MDI-treated (only 26% on pumps). Both cohorts were at the less favorable end of the outcome spectrum, which is consistent with the previous evidence that CSII confers modest advantages in GV and severe hyperglycemia during Ramadan fasting [10].

Regression to the Mean and Baseline Control

The fact that trials with the worst baseline glycemic control had the most apparent space for improvement or decline is another factor contributing to the disparity. The cohort of Al Hayek and Al Dawish started out with a markedly elevated GRI (56.4) and CHyper (21.0), indicating poor baseline control. The significant "improvement" during the structured, closely monitored fasting month may partially reflect regression toward better control under intensified short-term supervision rather than a long-lasting physiological benefit of fasting per se; this is further supported by the near-complete reversion of GRI and TIR within a month after Ramadan. In contrast, persons with superior baseline control (HbA1c ≤7%) in Surrati et al. showed little, temporary change, whereas those with weaker control (HbA1c >7%) experienced the greatest, longest-lasting decline-the identical subgroup effect acting in the opposite direction.

What is consistent across studies?

Hypoglycemia Risk Did Not Rise During Fasting

None of the four CGM-based studies reviewed here found a worsening of TBR or hypoglycemia-related metrics during Ramadan, despite the historical framing of Ramadan fasting as primarily a hypoglycemia risk period - a framing rooted in the EPIDIAR - era literature describing a 4.7-fold increase in severe hypoglycemia among fasting patients with T1DM. TBR remained constant (around 3% throughout), according to Surrati et al. In their last study year, Alguwaihes et al. discovered that TBR decreased (rather than rose) during Ramadan. During fasting, Al Hayek and Al Dawish observed substantial decreases in TBR and CHypo at the <54 and 54-69 mg/dL thresholds. Hypoglycemia did not significantly change at either severity level, according to Teoh et al. This consistent pattern across four independent, geographically and demographically different cohorts indicates that hyperglycemia, rather than hypoglycemia, is the predominant and more variable metabolic risk of Ramadan fasting in T1DM in the CGM era, with its capacity for closer monitoring and, in several cohorts, structured pre-Ramadan insulin adjustment. Surrati et al. and Alguwaihes et al. also explicitly noted this shift in their discussion.

Hyperglycemic Excursions Clustered Around Iftar and Suhoor

The detailed time-of-day analysis by Alguwaihes et al. is especially instructive: pooled 24-hour glucose profiles revealed the lowest average glucose in the mid-afternoon (14:00-16:00 h, a window the authors identify as the period of greatest hypoglycemia risk during fasting hours), followed by a sharp rise after Iftar (peaking around 20:00-22:00 h) and a second sharp rise after Suhoor (peaking around 4:00-6:00 h). Crucially, non-diabetic control subjects who fasted during Ramadan 2022 showed similar diurnal pattern, with a peak after Iftar and a nadir before, but their glucose levels stayed within the normal range the entire time. The cultural and dietary customs surrounding Iftar and Suhoor (large, high-fat and carbohydrate meals, food bazaars, sweetened beverages, and disturbed sleep/cortisol rhythms) are implicated in this parallel pattern in individuals without diabetes as a shared physiological driver, superimposed on - and probably compounding - inadequately adjusted insulin dosing in individuals with T1DM. This finding reframes the clinical goal: the time, makeup, and insulin coverage of the Iftar and Suhoor meals are what most directly affect glycemic outcomes, not fasting per se.

Acute Complications Remained Rare

Despite significant CGM-detected deterioration in some cohorts, it is encouraging that none of the studies reporting on acute complications documented episodes of diabetic ketoacidosis or hospitalization for acute hyperglycemic or hypoglycemic emergencies during the Ramadan study period (Surrati et al.; Al Hayek and Al Dawish). This implies that even in cases where daily glycemic control is not perfect, fasting can typically be carried out without acute life-threatening events with proper pre-Ramadan risk stratification along DAR/IDF-DAR criteria [11]. All four studies excluded very-high-risk patients (e.g., recent DKA, hypoglycemia unawareness, pregnancy, or insufficient CGM wear time).

Post-Ramadan persistence: a neglected window

Of the four investigations, only two (Alguwaihes et al. and Al Hayek and Al Dawish) properly evaluated the post-Ramadan period, and both discovered that the glycemic alterations seen during fasting did not last. According to Alguwaihes et al., the window of clinical risk extended beyond the actual fasting month because the aberrant post-Iftar and post-Suhoor glucose elevations continued for at least a month after Ramadan concluded. Al Hayek and Al Dawish discovered the opposite direction of change but a similar lack of durability: within a month, the significant in-Ramadan gains in GRI, CHypo, CHyper, and TIR mostly returned to pre-Ramadan levels, suggesting a return to less regimented eating and insulin-dosing practices. Altogether, these results show that any change in glycemic control during Ramadan is brittle and reflects the structured, transient conditions of the fasting month rather than a long-term change in disease management. This emphasizes the significance of the ongoing post-Ramadan follow-up and support, a dimension that is specifically advised but has not been thoroughly examined in the current body of evidence.

Role of CGM technology and emerging metrics

FreeStyle Libre-family sensors were used in all four investigations; however, their modalities varied: Alguwaihes et al. used flash glucose monitoring (FGM) evaluated through the LibreView platform, Surrati et al. and Al Hayek and Al Dawish used patient-facing intermittently scanned CGM (isCGM), and Teoh et al. used a retrospective, professional ("blinded") CGM that patients were unable to view in real time. This heterogeneity is important because, while fully retrospective CGM (as in Teoh et al.) offers useful descriptive data but no chance for in-the-moment behavioral or insulin-dosing correction, it likely biases that cohort toward smaller observed differences regardless of the true underlying risk. In contrast, real-time or scan-visible CGM enables patients to react quickly to trends.

The GRI, a composite measure that combines CHypo and CHyper components into a single score, was employed by Al Hayek and Al Dawish. This methodological advancement was not employed in the other three studies. When it comes to selecting patients that require greater attention, GRI provides a single, clinically interpretable value that may be more sensitive to overall risk than TIR alone. Its omission from the other three studies gives a chance for future harmonization of CGM-derived Ramadan outcome reporting and restricts direct cross-study comparison.

Limitations of the current evidence base

The conclusions that can be made from these four studies are tempered by a number of methodological issues. First, none of the studies are randomized controlled trials, and none have a non-fasting T1DM comparator group (Alguwaihes et al.'s non-diabetic fasting controls are informative for isolating the effect of meal timing and composition but cannot isolate the effect of fasting itself in T1DM). Second, sample numbers for important subgroup analyzes were frequently small, limiting the accuracy and generalizability of subgroup results. For example, Teoh et al.'s optimal-HbA1c subgroup included only four participants, while Surrati et al.'s HbA1c ≤7% subgroup had just 19. Third, attrition and selection bias were significant in the longitudinal cohorts: Surrati et al. excluded 40 of 121 consented participants (33%) due to inadequate CGM sensor wear time, and Alguwaihes et al. retained only 39 of 76 initially enrolled participants (51%) over a three-year period. These findings raise the possibility that included participants were consistently better resourced or more adherent than those excluded. Fourth, direct numerical comparison was complicated by the use of different glycemic thresholds and reporting conventions in these studies (e.g., Surrati et al. and Alguwaihes et al. used the standard 70-180 mg/dL TIR band, while Teoh et al. and Al Hayek and Al Dawish additionally reported level 1/level 2 hyperglycemia and hypoglycemia stratifications). Fifth, it is difficult to identify education as a causative mediator rather than just an associated marker of better-resourced care because none of the research standardized or controlled for the particular content of pre-Ramadan education. Lastly, the present review is deliberately limited to a summary of these specific four articles because they represent the current entirety of the literature that exclusively utilizes objective, continuous glucose monitoring (CGM) metrics to evaluate glycemic dynamics during Ramadan in type 1 diabetes, highlighting the critically small number of studies available on this topic.

Implications for clinical practice

Despite this variation, a number of practical implications are comparable throughout the four trials and are in line with current IDF-DAR recommendations. First, pre-Ramadan risk stratification is still crucial. All four studies limited participation to patients who did not meet extremely high-risk exclusion criteria, and this choice probably helped to prevent acute problems even in cases where day-to-day control deteriorated. Second, given its special capacity to identify the particular Iftar- and Suhoor-linked excursions that HbA1c and SMBG are unable to identify, CGM - ideally real-time or patient-visible rather than totally retrospective - should be regarded as the standard of therapy for individuals with T1DM who plan to fast. Third, the most consistent factor separating cohorts that maintained or improved glycemic control from those that declined was structured, ideally standardized, Ramadan-focused education given before the fasting month. This education covered individualized basal and bolus insulin retiming, meal composition advice for Iftar and Suhoor, and explicit thresholds for breaking the fast. Fourth, clinical attention should go beyond the actual month of fasting. In the two studies that evaluated the post-Ramadan period, glycemic changes in both directions proved to be temporary. To consolidate gains or reverse persistent hyperglycemic patterns, structured follow-up and reinforcement of self-management routines may be required in the weeks following Ramadan. Lastly, individuals who want to fast may benefit more from insulin pump therapy when it is available.

## Conclusions

A varied but illuminating picture of glucose control in TIDM during Ramadan fasting is presented by four recent, real-world CGM studies from Malaysia and Saudi Arabia. Glycemic control was either maintained or temporarily improved in situations where insulin pump therapy, younger age, and structured pre-Ramadan education were present; in adult cohorts where routine, individualized (as opposed to structured) care predominated, significant declines in time in range and average glucose were noted. Two results, however, were notably consistent across all four studies: hyperglycemia, which was concentrated in the hours after Iftar and Suhoor, emerged as the predominant and most modifiable risk, while hypoglycemia exposure did not rise during fasting. These results highlight the need for larger, harmonized, and ideally interventional studies, including standardized CGM metrics such as the GRI, to determine which particular components of structured care most consistently translate into long-lasting, rather than temporary, glycemic benefit. They also argue for reframing Ramadan diabetes care around structured education and CGM-guided, meal-timed insulin adjustment rather than around fasting avoidance alone.
